# Molecular mechanisms underlying the interaction between ZD1839 (‘Iressa’) and cisplatin/5-fluorouracil

**DOI:** 10.1038/sj.bjc.6601131

**Published:** 2003-07-29

**Authors:** N Magné, J-L Fischel, C Tiffon, P Formento, A Dubreuil, N Renée, J-L Formento, M Francoual, J Ciccolini, M-C Etienne, G Milano

**Affiliations:** 1 Centre Antoine Lacassagne, Oncopharmacology Unit, 33, Avenue de Valombrose, 06189, Nice cedex 2, France; 2 Pharmacokinetic Department, School of Pharmacy, Marseille, France

**Keywords:** ZD1839, ‘Iressa’, fluorouracil, cisplatin, head and neck cancer, molecular mechanisms

## Abstract

ZD1839 (‘Iressa’), an orally active, selective epidermal growth factor receptor (EGFR) tyrosine kinase inhibitor, is currently being investigated in clinical trials as a treatment for cancer. ‘Iressa’ is a trademark of the AstraZeneca group of companies. We have previously demonstrated a synergistic interaction between ZD1839 and cisplatin/5-fluorouracil (5FU) in CAL33, a human head and neck cancer cell line that markedly expresses EGFR. This study examined the effects of this drug combination on the cell cycle, cell cycle regulators, apoptosis-related factors, EGFR-related signalling and DNA repair in CAL33 cells. The cells were incubated with ZD1839 alone for 48 h, then cisplatin and 5FU were added. Exposure to the drug combination continued for a further 48 h. ZD1839 alone induced accumulation of cells in the G0/G1 phase of the cell cycle at 24 h accompanied by a concomitant increase in p21, p27 and Bax, a significant decrease in Bcl2 and a decrease in Akt phosphorylation. A decrease in DNA-PK was observed at 48 h. ZD1839 alone had no effect on caspase-3 activity, but addition of ZD1839 to cisplatin-5FU led to a significant increase in caspase-3 activity at 96 h. Thus, ZD1839 affects key cellular pathways controlling cell proliferation, apoptosis and DNA repair. These data provide a rationale to support clinical trials combining ZD1839 and cisplatin–5FU and other protocols that combine EGFR-targeting agents with chemotherapy or radiotherapy.

ZD1839 (‘Iressa’) is an orally active, selective epidermal growth factor receptor tyrosine kinase inhibitor (EGFR-TKI) that blocks signal transduction pathways implicated in the proliferation and survival of cancer cells and other host-dependent processes promoting cancer growth. ‘Iressa’ is a trademark of the AstraZeneca group of companies. It exhibits a broad spectrum of antitumour activity against many human solid tumour xenografts of various origins including breast, pancreas, lung, colorectal and head and neck (Ciardiello *et al*, 2000). ZD1839 has shown promising clinical effectiveness against a range of solid tumours, most importantly in non-small-cell lung cancer (Fukuoka *et al*, 2002; Natale *et al*, 2002). There are numerous experimental data both *in vitro* and *in vivo* showing that EGFR targeting is able to augment the antitumour activity of several anticancer agents including doxorubicin, cisplatin, 5-fluorouracil (5FU), gemcitabine, paclitaxel and topotecan (Baselga *et al*, 1993; Fan *et al*, 1993; Ciardiello *et al*, 1999; Bruns *et al*, 2000; Inoue *et al*, 2000; Overholser *et al*, 2000). Similar enhancement of activity has also been observed in studies combining EGFR-targeting agents, particularly the monoclonal antibody IMC C225 (cetuximab), with ionizing radiation (Huang *et al*, 1999; Saleh *et al*, 1999; Bianco *et al*, 2000; Bonner *et al*, 2000; Milas *et al*, 2000). Other investigators (Williams *et al*, 2002) and this laboratory (Magné *et al*, 2002a) recently showed supra-additive effects between irradiation and ZD1839, confirming the radiosensitisation conferred by an EGFR-targeting agent. We also reported a synergistic interaction between ZD1839 and the cisplatin–5FU combination (Magné *et al*, 2002a). This synergistic cytotoxicity observed *in vitro* in the head and neck human cancer cell line CAL33 was strictly dependent on the order of combination, with optimum effects observed when ZD1839 was applied before and during cisplatin–5FU treatment. The present study was undertaken to elucidate the cellular mechanisms underlying the synergistic cytotoxicity of the ZD1839 plus cisplatin–5FU combination.

Targeting EGFR signalling with specific drugs affects cellular pathways involved in cell cycle regulation, apoptosis and DNA repair (Ciardiello *et al*, 2000; Huang and Harari, 2000; Sirotnak *et al*, 2000; Baselga, 2001; Yarden, 2001). The following parameters were thus investigated in the CAL33 cell line exposed to ZD1839 and cisplatin–5FU: cell proliferation markers (cell cycle, p42/p44, p-AKT, p21, p27), apoptosis factors (p53, Bax/Bcl2, CD95, caspase 8, caspase 3) and DNA-repair mechanisms (DNA-dependent protein kinase (DNA-PK), ATM). Changes in glutathione *S* transferase *π* (GST*π*) expression were also investigated, as this is a key factor affecting the chemosensitivity to cisplatin (Cabelguenne *et al*, 2001).

## MATERIALS AND METHODS

### Chemicals

ZD1839 was kindly provided by AstraZeneca L, Rveic-Malmaison, France. Cisplatin and 5FU were provided by the pharmacy of the Centre Antoine Lacassagne (Nice, France). A 50 mM working solution in dimethylsulfoxide (DMSO) was prepared before use. Dulbecco's modification of Eagle's medium (DMEM) and glutamine were purchased from Whittaker (Verviers, Belgium). Fetal bovine serum (FBS) was obtained from Dutscher (Brumath, France). Penicillin and streptomycin were from Meyrieux (Lyon, France). Bovine serum albumin (BSA) and DMSO were purchased from Sigma (St Quentin Fallavier, France).

### Drug administration schedule

The human head and neck cancer cell line CAL33 was used in the present study as in our previous work (Magné *et al*, 2002a). CAL33 cells carry a p53 mutation (codon 175: CGC → CAC) and exhibit high EGFR expression (34 000 fmol mg^−1^ protein). CAL33 has no intrinsic mitogen-activated protein (MAP) kinase activity or k-ras mutation (Magné *et al*, 2002b).

Cells were routinely cultured in DMEM supplemented with 10% FBS, 2 mM glutamine, 50 000 UL ^−l^ penicillin and 80 *μ*M streptomycin, in a humidified incubator (Sanyo, Japan) at 37°C with an atmosphere containing 8% CO_2_.

Cells were seeded in 175 cm^2^ tissue culture flasks at 1.86 × 10^6^ cells to obtain exponential growth for the duration of the experiments. These conditions are comparable to 3000 cells per well in 96-well microtitration plates, as used in the previously published study (Magné *et al*, 2002a).

**Figure 1:**
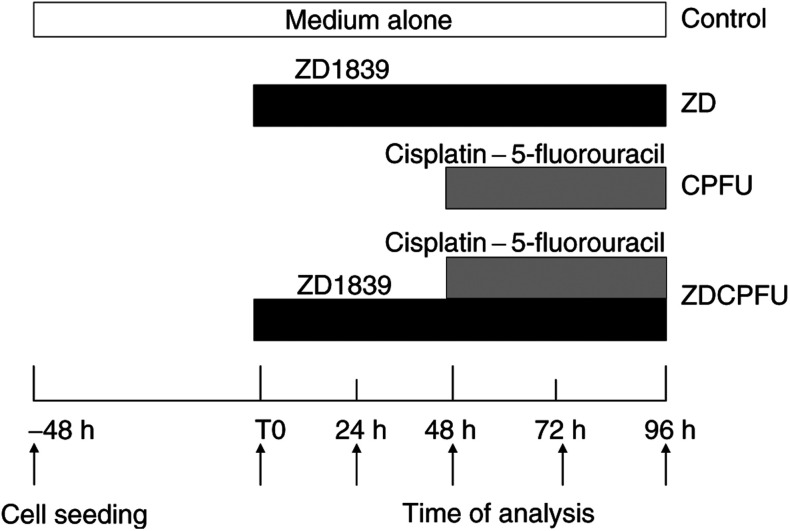
Study design. Single-drug exposures: 48 h after seeding, cells were exposed to ZD1839 alone for 96 h (condition ZD1839); 48 h after seeding, cells were exposed to medium alone for 48 h, followed by cisplatin–5FU for a further 48 h (condition CPFU). Drug combination: 48 h after seeding, CAL33 cells were exposed to ZD1839 (ZD) alone at its IC_50_ (6 *μ*M) for 48 h, then exposed to cisplatin and 5FU at their IC_30_'s in combination with ZD1839 for a further 48 h (condition ZDCPFU).

We investigated two different experimental schedules (Figure 1

; see below), and cellular parameters were analysed at 0, 24, 48, 72 and 96 h after T0, the onset of drug application.

#### Single-drug exposure

Cells were exposed, 48 h after cell seeding, to ZD1839 alone at 6 *μ*M (IC_50_) for 96 h (condition ZD1839); 48 h after seeding, cells were exposed to medium alone for 48 h, followed by cisplatin and 5FU together at their IC_30_'s (0.15 and 3.3 *μ*M, respectively) for a further 48 h (condition cisplatin–5FU; Figure 1). Cisplatin–5FU was considered to be a single drug for the purpose of this study.

#### Drug combination

CAL33 cells were exposed, 48 h after seeding, to ZD1839 at 6 *μ*M alone for 48 h. Cisplatin and 5FU (0.15 and 3.3 *μ*M, respectively) were then added, and exposure to the three-drug combination continued for a further 48 h (condition ZD1839–cisplatin-5FU) (Figure 1). The cytotoxic effects resulting from this drug combination were analysed as described previously (Magné *et al*, 2002a). A mean combination index (according to Chou and Talalay, 1984) of 0.4 was found, which confirmed the existence of the previously reported synergistic cytotoxic activity (Magné *et al*, 2002a).

All investigations were performed in triplicate during separate experiments for all cellular parameters.

### Cell cycle analysis

Cell cycles were analysed by FACS according to the Vindelov model. A total of 2 × 10^6^ CAL33 cells in exponential growth were treated with various concentrations of ZD1839 for 24 h and then washed three times with citrate buffer. The cell pellet was incubated with trypsin-containing citrate buffer (250 *μ*l) for 10 min at room temperature and then incubated with 200 *μ*l of citrate buffer containing a trypsin inhibitor and RNase (10 min) before adding propidium iodide (PI; 200 *μ*l at 125 *μ*g ml^−1^). Samples were analysed on a Becton Dickinson FACScan flow cytometer using Modfit software, which was also used to determine the percentage of cells in the different phases of the cell cycle. Propidium iodide was excited at 488 nm, and fluorescence was analysed at 620 nm (Fl-3).

### Western blot analyses

The following parameters were analysed by Western blotting: p21, p27, Bax, Bcl2, p-AKT, p42-p44, DNA-PK and ATM.

Western blots were performed by harvesting total cellular lysates (4 × 10^6^ cells) in 50 *μ*l of lysis buffer Laemmli 1 × (Laemmli 4 × : 1.6 ml Tris-HCl, 1 M, pH 6.8; 400 mg sodium dodecyl sulfate (SDS); 2 ml glycerol; 145 *μ*l *β*-mercaptoethanol; 12% bromophenol), and were heated for 15 min at 95°C. The protein content of cytosolic preparations was determined by the method of Bradford using the Bio-Rad reagent with BSA as a standard. Equal amounts of protein (50 *μ*g per lane) were separated by 12.5% SDS–10% polyacrylamide gel electrophoresis (PAGE) and transferred onto a nitrocellulose membrane. Prestained molecular weight markers were included in each gel. Membranes were blocked for 30 min in TBS (10 mM Tris-HCl, pH 7.5; 150 mM NaCl) with 0.5% Tween-20 and 5% BSA. After blocking, membranes were incubated for approximately 12 h with the appropriate specific anti-human monoclonal antibody (dilution of 1 : 5000) in TBS Tween and 1% BSA: p21 (mouse anti-human monoclonal anti-p21 antibody, Santa Cruz Biotechnology, Tebu Bio Le Perray en Yvelyne, France), p27 (mouse anti-human monoclonal anti-p27 antibody, Santa Cruz Biotechnology), Bax (mouse anti-human monoclonal anti-Bax antibody, Santa Cruz Biotechnology), Bcl2 (mouse anti-human monoclonal anti-Bcl2 antibody, Santa Cruz Biotechnology), phosphorylated form of AKT (p-AKT, goat anti-human polyclonal anti-p-AKT antibody, Santa Cruz Biotechnology), p42–p44 (mouse anti-human monoclonal anti-MAP kinase activated antibody, diphosphorylated ERK-1&2, anti-DPERK, clone MAP kinase-YT, Sigma, St Quentin Fallavier, France), DNA-PK (goat anti-human polyclonal anti-DNA-PK antibody, Santa Cruz Biotechnology) and ATM (mouse anti-human monoclonal anti-ATM antibody, Santa Cruz Biotechnology). After washing the membranes three times with TBS Tween (5 min each), they were incubated with peroxidase-conjugated secondary antibodies purchased from Dako (dilution of 1 : 1000; Glostrup, Denmark). A chemoluminescence reaction was performed and the membranes were exposed to ECL hyperfilm according to the manufacturer's instructions (Amersham Pharmacia Biotech, Little Chalfont, UK). Quantitative analysis of activities was performed by imaging the autoradiograms and quantitating relative band densities using scan imaging software (Image Master, Pharmacia, Amersham Bioscience, Orsay, France).

### p53 protein assay

p53 protein content (wild type and mutated forms) was analysed in cytosol (200 *μ*l) using a monoclonal two-site single incubation immunoluminometric assay (LIA-mat, Sangtec, Sweden). A total of 10^7^ cells were homogenised in lysis buffer (500 *μ*l; 50 mM Tris-HCl pH 6.8, 1% Triton X-100, 2 mM phenylmethylsulfonyl fluoride (PMSF) and 0.02% mercaptoethanol); the samples were then centrifuged at 105 000 **g** for 30 min at 4°C before 200 *μ*l aliquots were taken. The assay is based on the use of two monoclonal antibodies against different denaturing-resistant epitopes of the N-terminal part of the p53 protein. Polystyrene tubes were coated with PAb 1801 monoclonal anti-p53 antibody and the tracer consisted of isoluminol-conjugated D01 monoclonal antibody. The sensitivity limit was 0.002 ng mg^−1^ protein. The coefficient of variance for inter-assay reproducibility (pooled cytosol, *n*=10) was 7.66%.

### Detection of the CD95 (APO1-fas) receptor

In total, 10^6^ cells were trypsinised, washed and exposed to 4 ng *μ*l^−1^ of CH11 anti-Fas MAb for 45 min at 4°C. After two washing steps, cells were resuspended in DMEM containing 1:200 (v v^−1^) goat anti-mouse IgM (Immunotech, Marseille, France) and incubated for an additional 30 min at 4°C. Cells were then washed twice, and cell surface expression of CD95 was assessed by FACScan. Analysis was carried out on a flow cytometer (FACScan, Becton Dickinson) using Cell Quest Software. Cells exposed to goat anti-mouse IgM served as negative FITC control. Fas expression was defined as the fluorescence ratio of CH11 Fas MAb : isotype-matched negative control MAb. Relative Fas expression in untreated cells was considered to be 100%.

### Mitochondrial energisation

Evaluation of mitochondrial membrane permeability was determined as the retention of the fluorescence dye 3,3′dihexyloxacarbocyanine iodide (DiOC_6_(3); Sigma). At various intervals, 2 × 10^6^ cells were washed with PBS, trypsinised and then placed in DMEM medium. The mitochondrial transmembrane potential (ΔΨ_m_) was measured with the cationic lipophilic fluorochrome DiOC_6_(3). Cells were incubated at 37°C for 15 min in the presence of 40 nM DiOC_6_(3), then placed on ice before the addition of PI (10 *μ*g ml^−l^). The fluorochrome incorporation was immediately analysed using a Becton Dickinson FACScan flow cytometer at 488 nm (excitation wavelength). Propidium iodide was used in all samples to exclude dead cells from the analysis. DiOC_6_(3) fluorescence was recorded in Fl-1 and PI was recorded in Fl-3. The percentage of apoptotic cells was calculated from an Fl-1/Fl-3 scattergram.

### Activity of caspases 3 and 8

Caspase activity was measured fluorometrically in cell lysates, as recommended by the manufacturer (Enzyme Systems Products, Livermore, CA, USA), using an artificial substrate (AFC-138) that fluoresces when cleaved (excitation at 400 nm, emission at 505 nm).

In total, 4 × 10^6^ cells were stimulated as indicated and then incubated for 30 min at 4°C in lysis buffer (50 mM HEPES pH 7.5, 150 mM NaCl, 20 mM EDTA, 0.2% Triton X-100, 1 mM PMSF, 10 *μ*g ml^−1^ aprotinin). Lysates were cleared at 10 000 **g** for 15 min at 4°C. Protein (100 *μ*g) was incubated in a 96-well plate with 0.2 mM of Ac-DEVD-pNA specific for caspase 3 or caspase 8. Caspase activity was measured at 410 nm in the presence or absence of *N*-acetyl-Asp-Glu-Val-Asp-CHO (1 *μ*M). Specific caspase activity was expressed in Ac-pNA cleavage or released absorbance.

### GSTπ expression

GST*π* expression was assayed by reverse transcriptase–polymerase chain reaction (RT–PCR) on cell pellets (3 × 10^6^ cells) stored at −80°C. Total RNA was isolated using the RNA NOW kit from BIOGENTEX (OZYME, Montigny-le-Bretonneux, France) based on a method derived from Chomczynski and Sacchi (1987). RNA quality was checked by agarose gel electrophoresis. Quantification was performed by densitometric analysis at 260 nm. Total RNA (1 *μ*g) was preincubated for 5 min at 65°C in a 20 *μ*l final volume of 50 mM Tris-HCl (pH 8.3), 75 mM KCl, 3 mM MgCl_2_, 1 mM of each deoxyribonucleotide triphosphate and 2 *μ*M of random hexamers (Roche Diagnostics, Meylan, France). In all, 50 U Expand Reverse Transcriptase (Roche Diagnostics, Meylan, France) and 20 U human placenta ribonuclease inhibitor (Amersham Pharmacia Biotech, les Ulis, France) were then added and the mixture was incubated for 30 min at 42°C followed by 5 min at 94°C. The oligonucleotides used for GSTπ amplification were GST*π* sense strand: TAC ACC GTG GTC TAT TTC CC (nucleotides 27–46) and GST*π* antisense strand: CTG TTT CCC GTT GCC ATT GAT (nucleotides 627–647), which yield a 620 bp product. Those used for amplification of the reference gene (GAPDH) were GAPDH sense strand: GGA AGG TGA AGG TCG GAG TC (nucleotides 38–57) and GAPDH antisense strand: CAC AAG CTT CCC GTT CTC AG (nucleotides 218–237), which yield a 200 bp product. A LightCycler DNA Master SYBR Green I kit (Roche Molecular Biochemicals, Meylar, France) was used to perform GST*π* RT–PCR on the LightCycler apparatus. The kit contains MgCl_2_ 25 mM, LightCycler DNA Master SYBR Green I 10 × including deoxynucleotide triphosphate mix, MgCl_2_ 10 mM, SYBR Green I dye and Hot Start *Taq* DNA polymerase. cDNA (2 *μ*l), previously obtained, was amplified in duplicate (one incubation for GST*π*, one incubation for GAPDH, the control gene) in a 20 *μ*l reaction capillary containing 2 *μ*l of LightCycler DNA Master SYBR Green I 10 × , 1.6 *μ*l of 25 mM MgCl_2_ and 4 *μ*l of one pair of primers 2.5 *μ*M. In each experiment, a cDNA preparation of the RNA extracted from another CAL33 culture was introduced as the calibrator. Simultaneously, a blank was prepared as the negative control by incubating H_2_O PCR grade instead of cDNA. The PCR protocol programmed on the LightCycler software consisted of three steps. The first step was denaturation of cDNA and activation of the enzyme for 8 min at 95°C. In the second step, DNA was amplified for 45 cycles of 15 s at 95°C, 13 s at 61°C and 26 s at 72°C. In the third step, the temperature was raised gradually (0.1°C s^−1^) from the annealing temperature to 95°C to obtain melting curves and fusion temperatures of the amplicons synthesised. Results, calculated using RelQuant software, were expressed as the ratio of concentration of GSTπ to GAPDH for a given sample, normalised by the same ratio for the calibrator.

### Statistical analysis

Differences between the mean values were evaluated using either one-way ANOVA with Tukey's test or one-way ANOVA on ranks with Dunnetts' or Student–Newman–Keuls test, according to data distribution. *P*=0.05 was regarded as statistically significant. All analyses used SPSS software (Paris, France).

## RESULTS AND DISCUSSION

### Effects on cell proliferation (Figures 2, 3 and 9)

**Figure 9:**
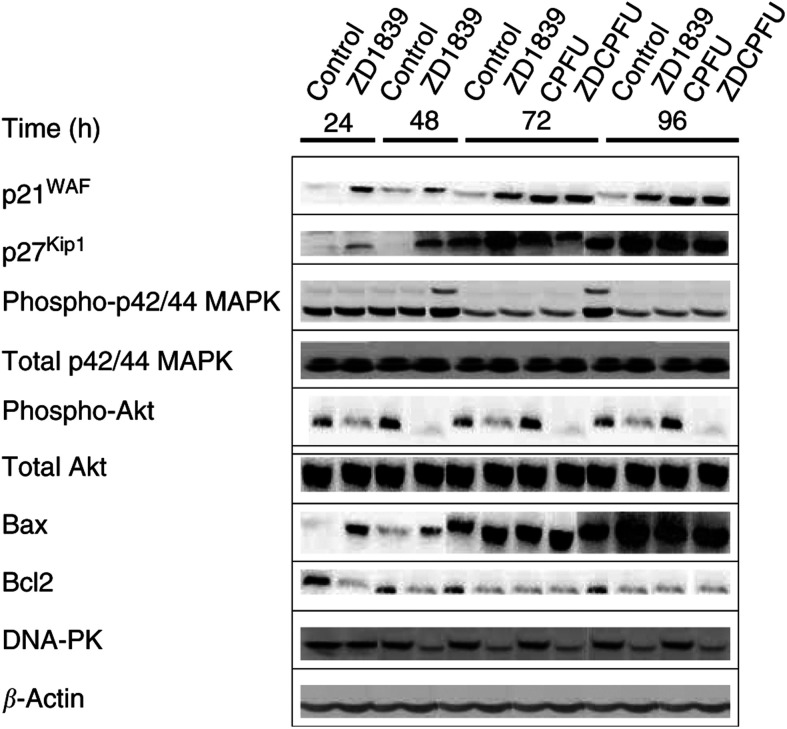
Representative Western blots related to Figures 2, 3, 4 and 8. For details on these analyses, see Materials and Methods section.

**Figure 2:**
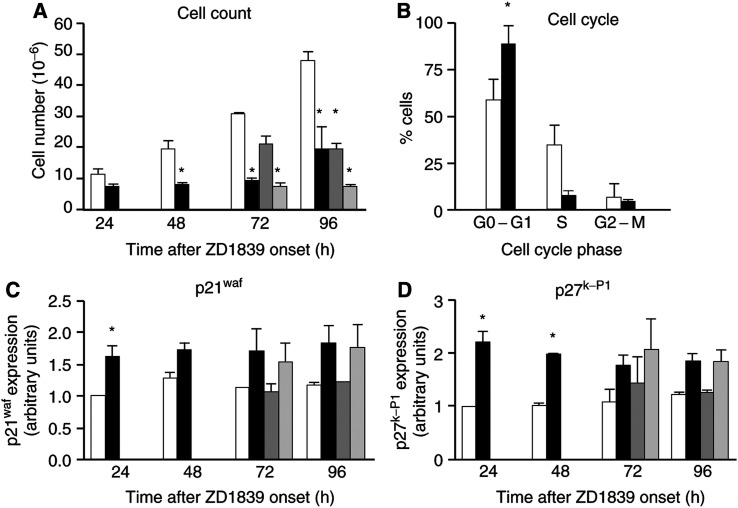
Impact of ZD1839 (6 *μ*M) and cisplatin–5FU (0.15 and 3.3 *μ*M, respectively) on cell proliferation and related molecular markers: (**A**) time-related impact of drugs on cell proliferation, (**B**) effect of ZD1839 on cell cycle at 24 h, (**C**) influence of drugs and drug combinations on p21 and (**D**) influence of drugs and drug combinations on p27. White bars=controls; black bars=ZD1839; dark grey bars=cisplatin–5FU combination; clear grey bars=ZD1839–cisplatin–5FU combination. Combination schedule as indicated in the legend to Figure 1.

In the absence of drugs, CAL33 cell numbers increased approximately five-fold over 96 h. Figure 2A

shows that ZD1839 markedly inhibited cell proliferation in a time-dependent manner. At the end of the longest drug exposure tested (96 h), ZD1839 reduced the final cell number by 60%; cisplatin–5FU had a similar effect. The combination of ZD1839 and cisplatin–5FU reduced the final cell number at 96 h six-fold compared with untreated cells. This degree of decrease in cell numbers suggests at least additivity. Previously, we have reported synergy when combining ZD1839 and cisplatin–5FU under identical experimental conditions (Magné *et al*, 2002a).

Flow cytometry analysis showed that the application of ZD1839 led to an accumulation in the percentage of cells in the G0/G1 phase compared with control (25% higher, maximal effect at 24 h, Figure 2B) and this result was not dependent upon exposure time. Fewer cells exposed to ZD1839 were in the S phase, regardless of the duration of exposure. The cell cycle effects of ZD1839 and the cisplatin–5FU combination were quite different: the latter treatment generated a greater proportion of cells gathered in the S phase. It is interesting to note that the final cell cycle effects when ZD1839 was combined with cisplatin–5FU were very similar to those observed after ZD1839 alone. The fact that ZD1839 enhances the proportion of cells in the G1 phase may favour the effect of 5FU through incorporation into RNA, as previously pointed out by Maybaum *et al* (1980).

Cyclin-dependent kinase (CDK) inhibitors are proteins that regulate the activities of CDK/cyclin complexes during the cell cycle. Many of the identified inhibitors, such as p21^WAF1^ and p27^Kip1^, act on G1-dependent kinases. ZD1839 exposure led, in CAL33 cells, to early increased expression of p21^WAF1^ and p27^Kip1^ with a maximum increase of 1.5- and two-fold, respectively, after 24 h (Figure 2C and D). These effects of ZD1839 on p21^WAF1^ and p27^Kip1^ agree well with those observed on the cell cycle. The present data obtained with ZD1839 are in line with previous data on tumour cells of head and neck origin concerning the upregulation of p27^Kip1^ caused by EGFR targeting with a specific monoclonal antibody (Huang *et al*, 1999). The cisplatin–5FU combination did not modify the cellular levels of these CDK inhibitors and no modulatory effect was observed in association with ZD1839.

**Figure 3:**
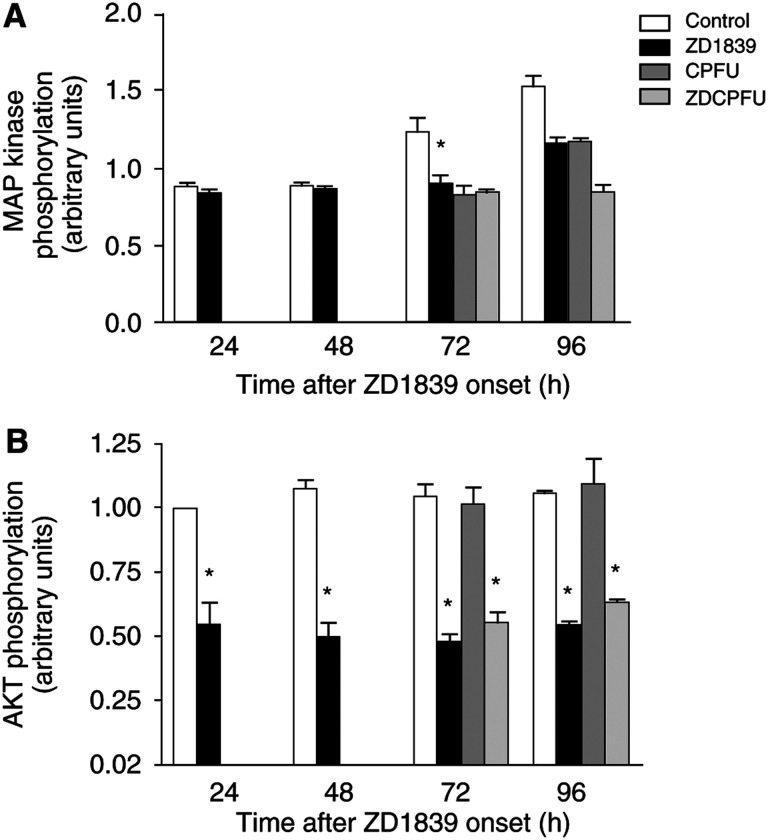
Impact of ZD1839 on key pathways regulated by EGFR signalling. (**A**) ZD1839 (6 *μ*M, IC_50_ value) diminished MAP kinase phosphorylation. (**B**) ZD1839 (6 *μ*M, IC_50_ value) diminished AKT phosphorylation. ^*^P<0.05 *vs* control, bars indicate standard deviation from the mean of three separate experiments.

We next examined the effects of ZD1839 on the final steps of EGFR signalling, p42/p44 (activated MAP kinase) and AKT (PI3 kinase pathway). Treatment of CAL33 cells with ZD1839 significantly reduced the level of phosphorylation of both MAP kinase and AKT (Figure 3A and B

). Importantly, ZD1839 reduced p42/p44 levels by 25% at 72 and 96 h, and it is interesting to note that these inhibitory effects were more marked (approximately a reduction of two-fold) and occurred earlier on AKT phosphorylation than on p42/p44. The impact of cisplatin–5FU on p42/p44 was comparable to that observed in cells treated with ZD1839 alone. In contrast, treatment with cisplatin–5FU did not induce marked changes in AKT phosphorylation. For both p-MAP kinase and p-AKT, the combination of cisplatin–5FU with ZD1839 did not generally lead to greater effects than cisplatin–5FU alone or ZD1839 alone. These observations support the recent data reported by other investigators showing that EGFR antagonists deplete the activation of both PI3 kinase and MAP kinase in human head and neck cancer cell lines (Bancroft *et al*, 2002) and human breast cancer cell lines (Normanno *et al*, 2002).

### Effects on apoptosis (Figures 4, 5, 6, 7 and 9)

**Figure 4:**
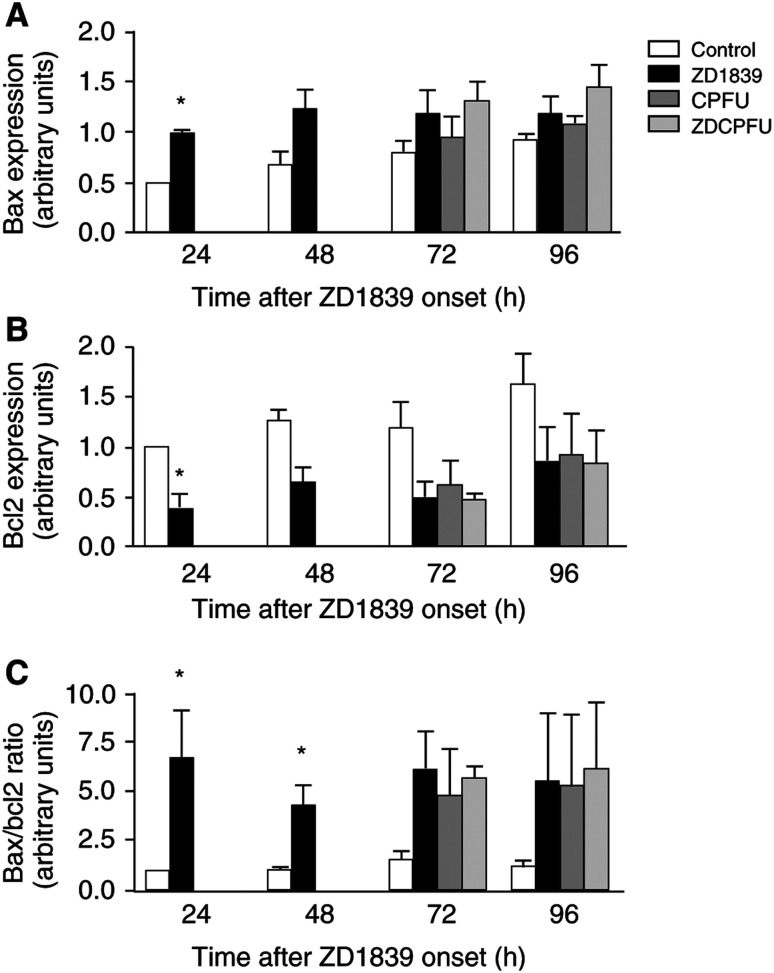
ZD1839 and cisplatin–5FU alone caused an upregulation of Bax (**A**) and a downregulation of Bcl2 (**B**), and no additive effect was observed when ZD1839 was combined with cisplatin–5FU. Results confirmed by the Bax/Bcl2 ratio (**C**). ^*^P<0.05 *vs* control, bars indicate standard deviation from the mean of three separate experiments.

As concerns the exploration of parameters linked to apoptosis, there are two different well-identified pathways. First, the mitochondrial route, which reacts to various stimuli of cell aggression (internal or external). This apoptotic pathway is initiated by a change in mitochondrial permeability, which is regulated by Bcl2-related proteins, and more particularly by the Bax/Bcl2 ratio, which is under the control of p53; the mitochondrial efflux in cytochrome C leads to the immediate activation of caspase 9 followed by the activation of the effector caspase 3. This later caspase catalyses the degradation of various proteins linked to vital cellular processes. Since a Bax/Bcl2-mediated pathway has been implicated in the response to effective drug treatment, we examined the changes in these pro- and antiapoptotic proteins in the presence of each agent. As shown in Figure 4A and B

, ZD1839 alone caused a downregulation of Bcl2 and an upregulation of Bax; both changes were already evident 24 h after treatment. Cisplatin–5FU had similar effects. As a corollary, ZD1839 alone caused an early and marked increase in the Bax/Bcl2 ratio maintained during all drug exposure and without modulatory effect caused by cisplatin–5FU (Figure 4C). This biochemical event likely reflects the capacity of EGFR targeting to upregulate the intrinsic apoptotic capacity of treated cells, as has been demonstrated by others (Ciardiello *et al*, 2002).

**Figure 5:**
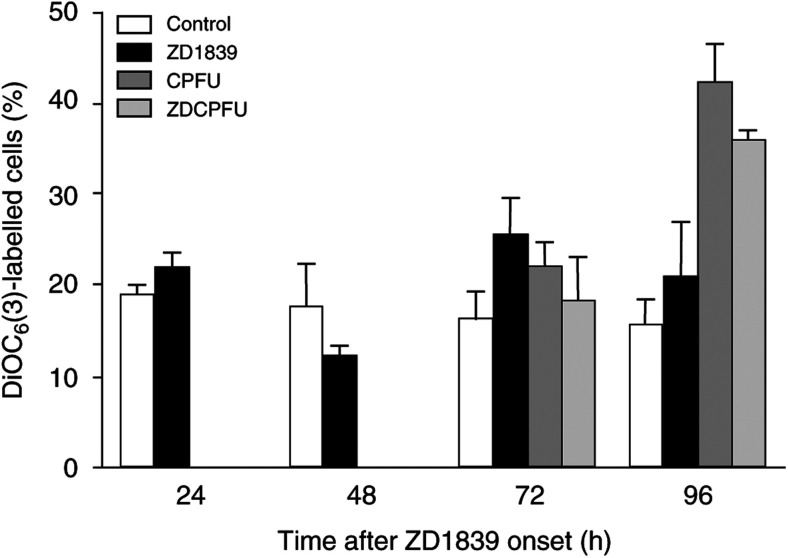
Impact of the applied drugs on mitochondrial energisation.

It is well established that, at an early stage, apoptotic stimuli alter ΔΨ_m_. We used the ampholytic cationic fluorochrome DiOC_6_(3) to monitor the changes in ΔΨ_m_ induced by ZD1839. Results are shown in Figure 5

. Cells treated for different lengths of time exhibited differences in fluorescence intensity. After 48 h of ZD1839 exposure, the maximum decrease in cellular uptake of the fluorochrome measured with the DiOC_6_ probe reflects the collapse of ΔΨ_m_, which is a signature for the opening of the mitochondrial megachannels, also called the permeability transition pores, in favour of involvement of the mitochondrial apoptotic pathway. In contrast, we observed an increase in DiOC_6_(3) fluorescence after 72 and 96 h of drug exposure, whatever the applied drug, although the Bax/Bcl2 ratio remained high.

Apart from the mitochondrial route, the cell death signal can be mediated through a receptor–ligand interaction via a membrane-linked death receptor family represented by tumour necrosis factor (TNF) and Fas. Fas (APO-1/CD95) is a member of the TNF receptor superfamily and can induce apoptosis when activated by its ligand FasL or by agonistic antibodies. The signalling cascade is initiated through the formation of a membrane death-inducing signalling complex that involves the recruitment of Fas-associated death domain protein and caspase 8. Neighbouring caspase 8 molecules within this newly formed multiprotein complex allow proteolytic autoactivation and the subsequent cleavage of downstream effector caspases (caspases 3, 6 and 7), leading to cell death. The impact of ZD1839 with or without cisplatin–5FU on Fas receptor expression and on caspase 8 activity was examined. Neither ZD1839 nor cisplatin–5FU had an impact on Fas receptor expression whatever the time of exposure (data not shown). This result is surprising because it has recently been shown that 5FU induces apoptosis, possibly via the Fas pathway (Backus *et al*, 2001). It seems, however, that Fas-mediated apoptosis stimulated by 5FU is dependent upon wild-type p53 status (Petak *et al*, 2000), a condition that is not fulfilled by the CAL33 cell line used in the present study. The lack of impact of ZD1839 on the death receptor pathway was confirmed by the absence of caspase 8 activity, whatever the applied drug and time of exposure (data not shown).

**Figure 6:**
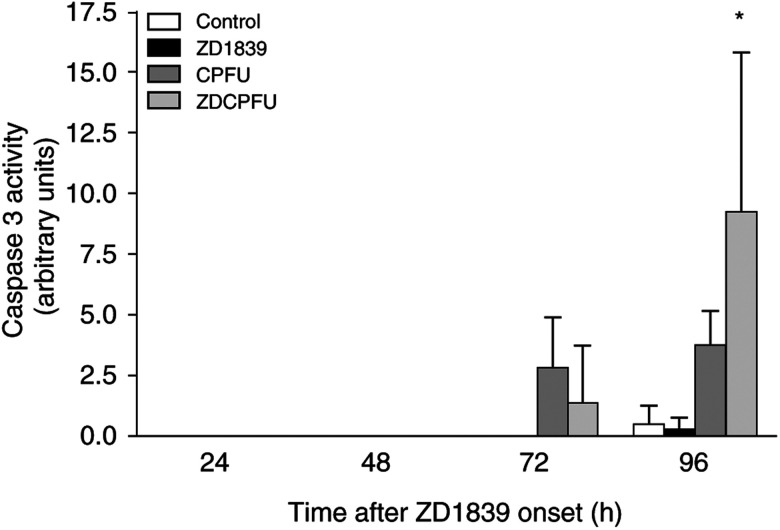
Impact of drugs and drug combination on caspase 3 activity. ZD1839 alone had no effect on caspase 3 activity, but the combination of ZD1839 with cisplatin–5FU led to a supra-additive increase in caspase 3 activity at 96 h. ^*^P<0.05 *vs* CFPU combination, bars indicate standard deviation from the mean of three separate experiments.

Results concerning caspase 3 were more informative. Interestingly, ZD1839 alone had no effect on caspase 3 activity, but the addition of ZD1839 to cisplatin–5FU led to a marked increase in caspase 3 activity at 96 h (Figure 6

). Recently, caspase 3 activation has been shown to play an important role in cisplatin-induced apoptosis and to precede the morphological changes typical to apoptosis (Kolfschoten *et al*, 2002). We feel the supra-additive increase in caspase 3 activity in the presence of ZD1839 and cisplatin–5FU is a molecular illustration of the synergistic interaction observed with this drug combination (Magné *et al*, 2002a). It is also interesting that ZD1839 alone had a minor effect on caspase 3 activity (only visible at 96 h) despite the fact that this drug was able to markedly upregulate the Bax/Bcl2 ratio. This later observation may be considered as a biochemical explanation of the radio- and chemo-sensitisation provided by EGFR-targeting drugs like ZD1839, which would be sustained by an underlying mechanism of enhancement of the intrinsic cellular apoptotic capacity.

**Figure 7:**
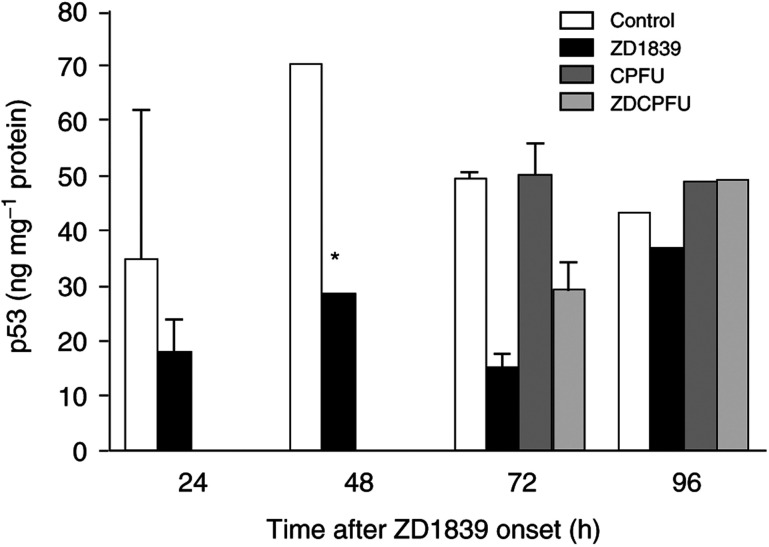
Impact of drugs and drug combination on p53. ^*^P<0.05 *vs* control, bars indicate standard deviation from the mean of three separate experiments.

It was striking to note that ZD1839 alone was able to markedly diminish p53 expression at 24–72 h with a maximum 2.5-fold decrease at 48 h; the inhibitory effect was smaller at 96 h (Figure 7

). Moreover, cisplatin–5FU alone or in association with ZD1839 had strictly no effect on p53 expression compared with controls at 72–96 h. Since changes in Bcl2 and Bax are in a great part under the control of p53 (Johnstone *et al*, 2002), one would expect the increase in Bax and the concomitant decrease in Bcl2 under ZD1839 to be accompanied by an upregulation of p53 levels in the presence of ZD1839. The same deduction holds true for the changes in p21 and those expected for p53. We have no clear explanation for these apparent contradictions; the present cell line was p53 mutated; this fact must be taken into consideration and it is plausible that this p53 status may influence the regulation of this protein under ZD1839. Thus, the fact that EGFR targeting modifies p53 cellular status merits further investigation.

### Effects on DNA repair and detoxification mechanisms (Figures 8 and 9)

**Figure 8:**
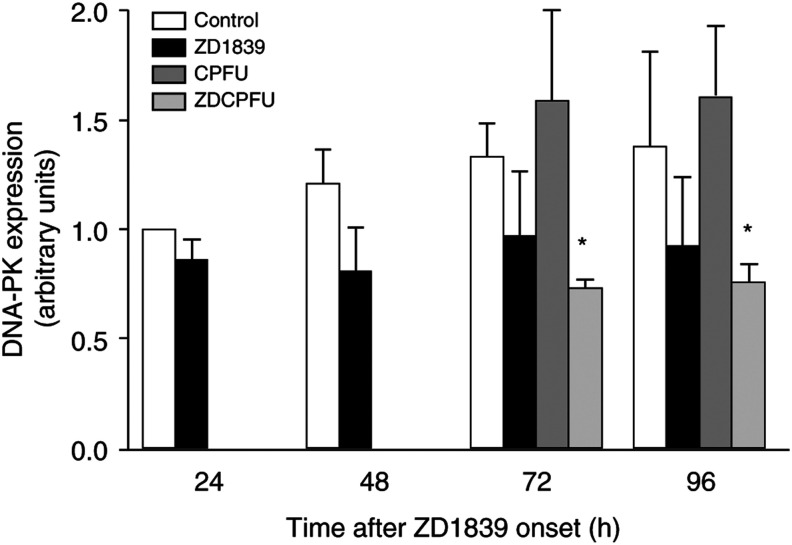
Impact of drugs and drug combination on DNA-PK. ^*^P<0.05 *vs* control, bars indicate standard deviation from the mean of three separate experiments.

DNA damage activates several protein kinases, of which the prototypes are ATM, mutated in the human autosomal recessive disorder ataxia telangiectasia, and DNA-PK. A target of ATM is the tumour suppressor p53, maintained at low levels through interaction with the MDM2 protein that signals p53 degradation. MDM2 is itself a target for DNA-PK. ZD1839 alone reduced DNA-PK expression by 25% compared with control. The application of cisplatin–5FU slightly increased DNA-PK expression (Figure 8

). The combination of ZD1839 and cisplatin–5FU significantly reduced DNA-PK expression at 72 and 96 h. There was no impact on the DNA-repair protein ATM whatever the exposure time or the drug applied. The impact of ZD1839 on DNA-PK expression may be one explanatory phenomenon for the synergistic interaction between ZD1839 and cisplatin–5FU, because DNA repair is one of the biochemical modulators of cisplatin sensitivity (Perez, 1998). Furthermore, DNA-PK is involved in the repair of radiation-induced DNA damage, suggesting a role for ZD1839 in radiosensitisation.

Glutathione, GSTπ and glutathione conjugate export pump have been shown to participate collectively in the detoxification of many anticancer drugs, including cisplatin (Perez, 1998). ZD1839 upregulated GST*π* expression after 24–72 h of exposure, with a maximum two-fold increase after 48 h. Similarly, cisplatin–5FU upregulated GST*π* at 96 h. The combination of ZD1839 and cisplatin–5FU did not lead to additional effects on GSTπ. In contrast to the impact of ZD1839 on DNA-PK, this latter observation is not consistent with a synergistic interaction resulting from combining ZD1839 with cisplatin–5FU.

In summary, the present experimental study brings together numerous biochemical arguments that may sustain not only chemo-sensitisation but also radio-sensitisation (impact on DNA-PK) conferred by EGFR targeting. ZD1839 was thus able to impact on key cellular pathways controlling cell proliferation, apoptosis and DNA repair. Although the observations were obtained on a single human cancer cell line, the present data may serve as a rational basis to support clinical trials combining ZD1839 and cisplatin–5FU and also other protocols that combine EGFR targeting with chemotherapeutic drugs or radiotherapy.
